# Systematic Review of Psychological Aging Research in Nepal

**DOI:** 10.1093/geroni/igab046.3641

**Published:** 2021-12-17

**Authors:** Kezang Tshering, Srijana Shrestha, Barbara Kamholz

**Affiliations:** 1 Wheaton College, Wheaton College, Massachusetts, United States; 2 Wheaton College, Norton, Massachusetts, United States; 3 UCSF, UCSF, California, United States

## Abstract

Nepal faces unprecedented levels of aging similar to trends in many less well-resourced countries. It has limited capacity to address the medical, social and psychological needs of older persons. Difficult choices regarding allocation of resources will be needed. In this review, we hope to clarify what is already known in aging research in Nepal. The databases APA PsychINFO and PubMed were searched. The inclusion criteria were peer-reviewed articles on i) psychological constructs and mental illnesses, ii) use of original data, iii) inclusion of senior participants and iv) studies conducted in Nepal. Studies that included mixed age group and cross-country comparisons were excluded from this review. The initial search resulted in 76 articles from APA PsychINFO and 590 articles from PubMed. Articles were reviewed independently for inclusion and exclusion criteria. A total of 49 articles were included in the final list. Preliminary results showed that the largest share of articles focused on depression (32.1%), followed by quality of life/life satisfaction or loneliness(18.9%). A large number of studies also examined prevalence rates of psychiatric and neurocognitive disorders (22.6%). Common conditions, like dementia and delirium were studied only in 1.9% and 3.8% of published studies respectively. All of the studies were cohort-based and none focused on evaluations of psychosocial/medical interventions. Robust intervention studies are needed to help improve the lives of seniors in Nepal. To our knowledge this is the first comprehensive review of published articles on psychological construct in aging populations in Nepal.

